# The effect of telerehabilitation on balance in stroke patients: is it more effective than the traditional rehabilitation model? A meta-analysis of randomized controlled trials published during the COVID-19 pandemic

**DOI:** 10.3389/fneur.2023.1156473

**Published:** 2023-05-17

**Authors:** Zhaoyin Su, Zhenxia Guo, Weitao Wang, Yao Liu, Yatao Liu, Wanqiang Chen, Maohua Zheng, Nerich Michael, Shuai Lu, Weining Wang, Handan Xiao

**Affiliations:** ^1^The First Clinical College of Medicine, Lanzhou University, Lanzhou, China; ^2^Department of Trauma Surgery, First Hospital of Lanzhou University, Lanzhou, China; ^3^Department of Anesthesia Surgery, First Hospital of Lanzhou University, Lanzhou, China; ^4^Department of Rehabilitation, First Hospital of Lanzhou University, Lanzhou, China; ^5^Department of Neurosurgery, First Hospital of Lanzhou University, Lanzhou, China; ^6^Department of Trauma Surgery, University Medical Center Regensburg, Regensburg, Germany; ^7^School of Stomatology, Lanzhou University, Lanzhou, China; ^8^The Second Clinical College of Medicine, Lanzhou University, Lanzhou, China

**Keywords:** COVID-19, stroke, telerehabilitation, meta-analysis, balance, rehabilitation, telemedicine

## Abstract

**Objective:**

Telerehabilitation and telemedicine have gradually gained popularity. In 2019, the outbreak of COVID-19 started in Wuhan and then spread across the world. To date, most countries have opted to coexist with the virus. However, patients, especially those who have suffered a stroke, should take measures to avoid being infected with any disease as much as possible since any infectious disease can lead to adverse events for them. Telerehabilitation can be beneficial to stroke patients as they are less likely to be infected by the virus. In recent years, several studies on telerehabilitation have been conducted globally. This meta-analysis aimed to investigate the effects of telerehabilitation on the balance ability of stroke patients, compare the efficacy of conventional rehabilitation with telerehabilitation, explore the characteristics of telerehabilitation and conventional rehabilitation, and provide recommendations for rehabilitation programs in the context of the global pandemic.

**Methods:**

We searched Pubmed, Embase, the Web of Science, and The Cochrane Library databases from 1 January 2020 to 31 December 2022 for randomized controlled trials published in English that evaluated the improvement of balance function in stroke patients after telerehabilitation and compared the differences between telerehabilitation (TR) and conventional rehabilitation (CR). The random-effects model was utilized to calculate mean differences (MDs) with 95% confidence intervals (CIs) to estimate intervention effects. Statistical heterogeneity was assessed according to the I^2^ values. The risk of bias was measured using the Cochrane risk-of-bias assessment tool.

**Results:**

We included nine studies in the system evaluation, all of which were included in the pooled analysis. All outcomes in the experimental and control groups improved over time. The comparison between groups concluded that people who received the telerehabilitation intervention had a significant improvement in the Berg Balance Scale (MD = 2.80; 95% CI 0.61, 4.98, *P* < 0.05, I2 = 51.90%) and the Fugl-Meyer Assessment (MD = 8.12; 95% CI 6.35, 9.88, *P* < 0.05, I2 = 0) compared to controls. The Timed Up and Go test (MD = −4.59; 95% CI −5.93, –.25, *P* < 0.05, *I*^2^ = 0) and Tinetti Performance-Oriented Mobility Assessment—Balance (MD = 2.50; 95% CI 0.39, 4.61, *P* < 0.05) scored better in the control group than in the experimental group. There were no significant differences in other outcomes between the two groups.

**Conclusion:**

Studies on changes in medical conditions during the COVID-19 pandemic also demonstrated that, for stroke patients, telerehabilitation achieves similar effects as the conventional rehabilitation model and can act as a continuation of the conventional rehabilitation model. Owing to the different equipment and intervention programs of telerehabilitation, its curative effect on the static balance and reactive balance of stroke patients may be different. Currently, telerehabilitation may be more conducive to the rehabilitation of patients' static balance abilities, while conventional rehabilitation is more effective for the rehabilitation of patients' reactive balance. Therefore, further studies are needed for investigating the difference in efficacy between varied devices and telerehabilitation programs. Further research is needed on static and reactive balance. In addition, such research should have a large body of literature and a large sample size to support more definitive findings based on the context of the COVID-19 pandemic.

**Systematic review registration:**

CRD42023389456.

## Introduction

Coronavirus disease 2019 (COVID-19) is caused by severe acute respiratory syndrome coronavirus 2 (SARS-COV2). The rapid spread of COVID-19 has led to major challenges to the world since it was first detected in Wuhan, China, at the end of 2019 (1, 2). By 12 January 2023, the number of confirmed cases related to COVID-19 had exceeded 660 million, including a staggering 6.69 million deaths (3). As of the writing of this article, the associated diagnoses and deaths were higher as the world's most populous country, China, has opened up. The morbidity and mortality of COVID-19 are much higher than those of common influenza, and the infection can cause persistent symptoms such as headache, fatigue, and dyspnea, which is called long COVID-19 (4–6). Every infection with COVID-19 causes great harm to the human body, and infection with COVID-19 is more likely to lead to adverse events for stroke patients or other people who are already affected by some type of disease. Given that COVID-19 is a highly contagious disease, medical facilities can be a source of infection, and new methods to avoid face-to-face contact between medical staff and patients are urgently needed (7). In the current situation, telemedicine has become one of the important options for providing medical services that can reduce the possibility of patients being infected with the virus by reducing face-to-face contact (8, 9). Telerehabilitation is the delivery of rehabilitation services to patients at a distance through information and communication technologies (10, 11). Remote communication between patients and physical therapists or rehabilitation professionals can occur through a variety of media, such as phone calls, text messages, Internet apps, Internet-based video conferencing, or virtual reality programs (12, 13), which enables rehabilitation services to be delivered over the Internet, effectively reducing patient visits to hospitals, costs, and the likelihood of infection. Balance is a complex function that encompasses dynamic balance and static balance (14). It is a major determinant of community ambulation and gait performance following strokes (15). The main obstacle to independence in daily living for stroke patients is the impairment of balance caused by the stroke (16). Approximately 75% of individuals with stroke in China have motor dysfunction, and 40% of them have a severe disability (17). Stroke survivors often have deficits in motor control, resulting in decreased balance (18, 19). Good motor control enables the body to maintain an upright posture to maintain balance; poor posture control will adversely affect the body's balance (20). Decreased static and dynamic balance is a major risk factor for falls in stroke patients (21, 22) and limits their ability to perform activities of daily living (23). They often lose their balance due to balance disorder, which leads to serious injury (24, 25). Therefore, one of the main goals of stroke rehabilitation is to restore the patient's functional balance (26), and the restoration of postural control is a prerequisite for the patient to perform activities of daily living independently (27). Given the importance of balance in the prognosis of stroke survivors and because we consider that the meta-analysis should be more fine-grained in the area of stroke rehabilitation to yield greater clinical significance, we chose to conduct the study from the perspective of balance in stroke patients rather than assessing the various aspects of change in stroke patients as a result of telerehabilitation. Despite the importance of balance function in stroke patients, previous studies remain incomplete and limited by traditional rehabilitation programs (28), making it important to investigate the effects of telerehabilitation on balance function in stroke patients. In recent years, several randomized controlled trials (RCTs) have been conducted to compare the effects of telerehabilitation with conventional rehabilitation in patients after stroke (29). These studies have shown that telerehabilitation is equal to (9, 30) or superior to conventional rehabilitation in terms of improving balance function in stroke patients (31–33). Although there have been some studies on telerehabilitation before, the COVID-19 pandemic has greatly changed all aspects of people's lives worldwide and impacted the conventional diagnosis and treatment model. As a result, stroke survivors have limited opportunities to obtain outpatient rehabilitation treatment (33); therefore, the application and effect of telerehabilitation in patients after a stroke may be different from the past. Moreover, a lot of innovative findings have been published in recent years, and discoveries in related fields are updated. Besides, previous meta-analyses have not concluded whether telerehabilitation is superior to conventional rehabilitation (29). This study aimed to explore the benefits of telerehabilitation in the rehabilitation of balance function after stroke during the COVID-19 pandemic using a meta-analysis framework, to research the characteristics of the outcomes brought by the two types of rehabilitation, and understand which modality, telerehabilitation or conventional rehabilitation, is more beneficial for patients to finally provide a reference for the rehabilitation mode of patients during the pandemic. Moreover, the defects and deficiencies of related research were discussed in this study to provide a reference for further research.

## Methods

This meta-analysis followed the guidelines for Preferred Reporting Items for Systematic Reviews and Meta-Analyses (PRISMA) (Supplementary material 1) (34, 35). This review was registered with Prospero with the unique identifier CRD42023389456.

### Search strategy

We searched Pubmed, Embase, the Web of Science, The Cochrane Library, and the Joanna Briggs Institute databases for articles from 1 January 2020 to 31 December 2022. The framework of Population, Intervention, Comparator, and Outcome (PICO) was used to search for eligible studies with the search terms stroke (P), telerehabilitation (I), and postural balance (O). The detailed search strategy can be found in Supplementary material 2. In addition, we adapted the terminology to suit the requirements of each database. References to systematic reviews with similar research questions were also manually searched.

### Criteria for considering studies for this meta-analysis

The inclusion criteria were as follows: (1) the study was conducted in English; (2) the efficacy of telerehabilitation after stroke was evaluated using varied modes; (3) an RCT design was used; and (4) the literature was published between 1 January 2020 and 31 December 2022. Studies that were not randomized or designed with one arm were excluded, as were studies that examined the technical components of telerehabilitation systems. According to the above criteria, the literature was imported into Endnote and Excel and evaluated by four reviewers in two stages: first the title and abstract, and then the full text. If two reviewers did not agree, the other reviewer resolved the disagreement.

### Data extraction and management

To extract prespecified data, two independent reviewers used prefabricated Excel sheets, including the study title, first author information, year of publication, participants, experimental group, control group, intervention and control protocol, follow-up, and the results of outcomes. We used Engauge Digitizer 11.1 (45) to extract data from figures when quantitative data were not reported in text or Supplementary material. For missing data, we emailed the author requesting availability and indicated that we would acknowledge him accordingly. Data were extracted as the mean (SD) of change before and after treatment and then compared between groups. When these values were not given in the included studies, they were calculated using the equations in the new edition of the Cochrane Handbook for Systematic Reviews of Interventions (46).

### Risk of bias assessment for the included studies

The Cochrane collaborative tool (47) was utilized by two researchers independently for estimating the risk of bias in the included studies. High risk of bias, low risk of bias, and unclear risk of bias were used to classify the included studies, which addressed the following sources of bias: (1) selection bias (random sequence generation and allocation concealment); (2) performance bias (blinding of participants and outcome assessors); (3) loss bias (incomplete result data); (4) reporting bias (selective reporting); and (5) other sources of bias. Discrepancies were resolved by discussion or consultation with a third investigator.

### Statistical analysis and outcome interpretation

All data analyses and graphical displays were performed using the STATA17.0 software. Post-intervention means and standard deviations were entered into STATA by one author and checked by another author. The values of the post-intervention outcomes were combined. The mean difference (MD) and 95% confidence interval (CI) for all outcomes were calculated using a random effects model (48). Subsequently, the *P*-values were statistically tested. Heterogeneity was assessed visually by forest plots and *I*^2^ statistics. The sensitivity analysis was conducted for the most important outcome.

## Results

### Results of the research

**Figure 1:**
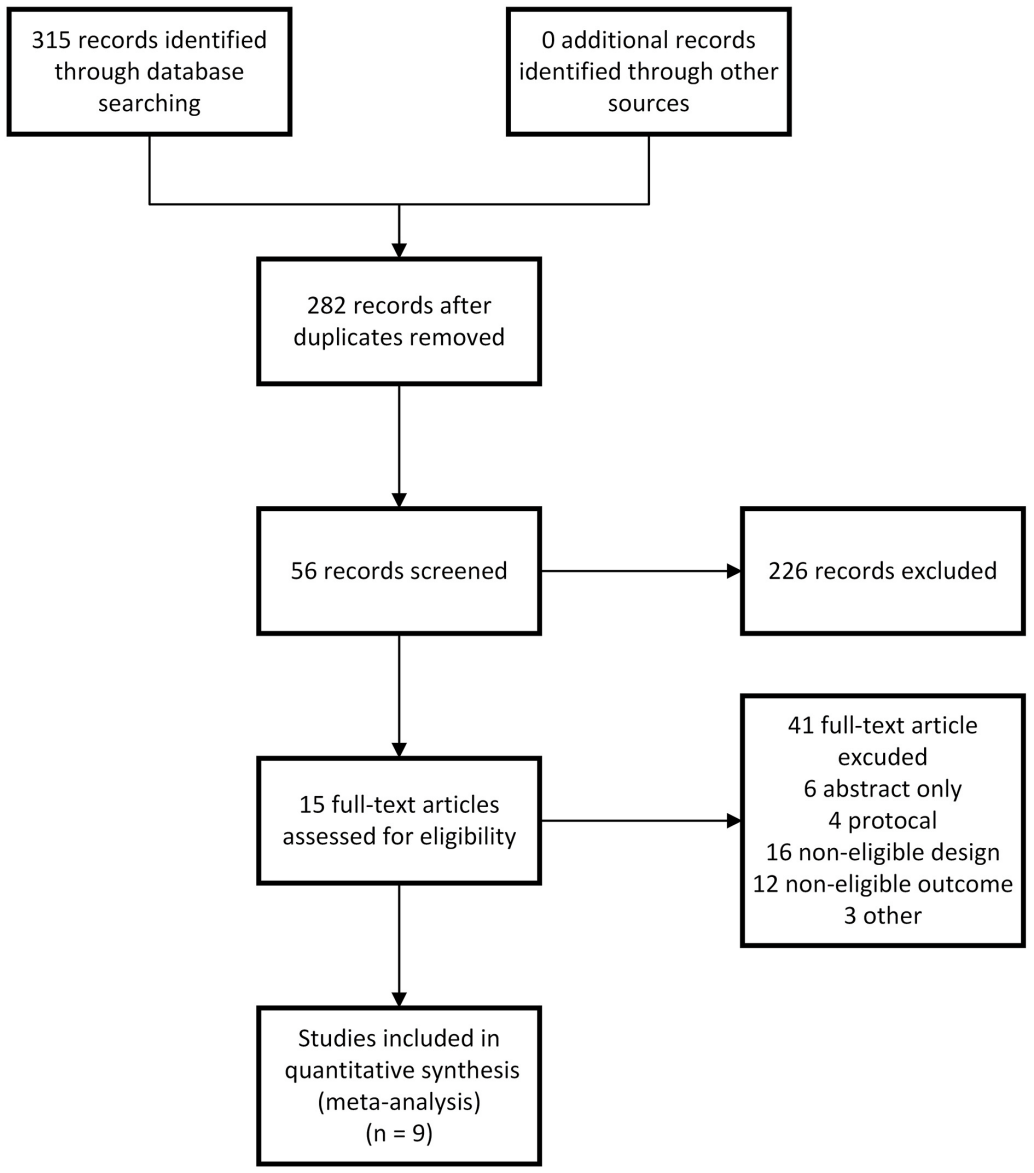
A flow diagram of the literature search and study selection procedure.

A total of 315 unique records were initially retrieved in our literature search, leaving 282 records after removing duplicates, which were reduced to 56 records after title and abstract screening. After careful full-text screening, nine RCTs (328 patients) (9, 30–33, 49–52) were identified to comply with the request of our systematic review and were included in the meta-analysis. Each study was published in English between 1 January 2020 and 31 December 2022. The flowchart of the search strategy and the study selection process is presented in Figure 1. Detailed search strategies are provided in Supplementary material 1.

### Characteristics of the included studies

The demographic characteristics, intervention modalities, outcomes, and main results of the included studies are provided in Table 1. The studies were published between 1 January 2020 and 31 December 2022. Overall, 328 patients were treated and evaluated, and the number of patients ranged from 17 to 61. All patients had a subacute or chronic stroke. Telerehabilitation interventions were varied (Nintendo Wii, Microsoft Kinect, or customized devices). Among them, Shih-Ching Chen et al. (30), together with Melisa Junata et al., used Microsoft Kinect (33), Elena Marques-Sule et al. utilized Nintendo Wii (32), and the intervention devices used in the remaining studies were customized. A control group was set up in all studies, except for Wu et al. (31), who added telephone follow-up with the conventional means of rehabilitation as the control group. In the other studies, there were only conventional forms of rehabilitation in the control group. All studies measured post-intervention outcomes in terms of time, and post-intervention scores were compared between groups in this review. The types of outcomes varied among the studies. The primary outcomes (Table 2) of the balance function were as follows: BBS, a 14-item balance scale that evaluates balance in different postures (53); TUG, expressed in terms of time, is a test that commonly evaluates the functional movement required for sitting, standing, and walking (54); S-TIS 2.0, a tool that evaluates dynamic sitting balance and trunk control in stroke patients (38); S-PASS, a 12-item scale (40) that assesses the ability to balance in three positions (lying down, sitting, and standing); S-FIST, a clinical functional assessment of sitting balance in adults with stroke (39); TIS, a scale that assesses static and dynamic sitting balance and trunk coordination (55); FMA, an assessment test that evaluates measures of limb movement, balance, sensation, joint range of motion, and pain (56); POMA, a 16-item scale (9 balance-related items and 7 gait-related items) (32); and ABC, a scale that estimates individual confidence in a sense of balance (57). A funnel plot was constructed for the most reported outcome in the included studies.

**Table 1:** Characters of study's include in this meta-analysis, k = 9.

**Reference**	**Patients (exp/ctrl)**	**Experimental**	**Control**	**Dosage**	**Outcomes**	**Key findings**
Chen et al. (30)	30 (15/15)	Telerehabilitation based on a Kinect camera-based interactive telerehabilitation system, including three commercially available video games, focusing on participants' balance, weight bearing, strength, weight shifting, and walking	CT, including sitting to standing movements, balance exercises, standing, overground walking, and facilitation or strengthening of the paretic limb. Therapists adjust the conventional physiotherapy according to the functional status of each participant	40 min/d, 3 d/w, 4 m	BBS, TUG	No significant differences were found
Salgueiro et al. (51)	49 (20/29)	APP plus CT. Users have been able to voluntarily access the exercises guide (description, photo and video) and to confirm its performance. Participants were asked to perform 10 repetitions of each of the 32 exercises proposed in the program and were encouraged to perform as many exercises as possible, respecting their perception of tiredness, taking as many breaks as they found necessary	CT, which consisted of face-to-face session of therapeutic techniques such as muscle stretching to reduce hipertonicity or spasticity, passive and functional mobilization of body segments affected by stroke, practice of sitting and standing posture and gait, task and aerobic training as cycling or treadmill training. The techniques used were chosen at the discretion of the physiotherapist in charge following the clinical practice guidelines. The intervention was totally adapted and personalized to the needs and capacities of the patient. Participants maintained their usual dose of treatment during participation in this study	20 min/d, 5 d/w, 3 m	BBS, S-TIS2.0, S-PASS, S-FIST	No significant differences were found
Salgueiro et al. (52)	30 (15/15)	Home-based core-stability exercises plus CT. The core stability training conducted during hospitalization was continued. All the training was made by experienced neurophysiotherapists and was monitored and talked through the APP	CT, including therapeutic techniques such as muscle stretching, passive and functional mobilization of the affected body segments, balance exercises and gait training	1 h/d, 2 d/w, 12 w	BBS, S-TIS2.0, S-PASS, S-FIST	Improvement in S-TIS2.0
Lee et al. (9)	17 (9/8)	40-min, non-face-to-face, dance-therapy plus CT. Dance classes begin with a warm-up while sitting, which transition into chair and/or standing choreography; which follows by dance-skill practice	CT, receiving conventional physical therapy for the duration the experimental group receives therapy (the dance program in addition to existing conventional physical therapy)	40 m/d, 2 d/w, 3 w	BBS, TUG, TIS	No significant differences were found
Junata et al. (33)	30 (16/14)	A Kinect-based Rapid Movement Training. Assessments for pre- and post-training included “lean-and-release” assessment and clinical score measures of balance confidence, balance, motor functioning, and independent mobility. The Kinect-based rapid movement training platform system prompted the RMT group participants with a limb (arm or leg) and a direction cue on a screen and tracked the 3D trajectory and timing of their movements. Participants were encouraged to perform arms and legs movement in 22 different directions as quickly and as far as possible. The 22 directions were randomized and were repeated four times	CT, the exercises include balance and functional weight-shifting training: sitting-to-standing (using a stool), lateral stepping (walk 3 m back and forth), forward and backward stepping (5 times right leg steps first and 5 times left leg steps first), forward walking for five meters (walk 3 m back and forth, turn right at one end and turn left at one end), stepping up and down (5 times right leg steps first and 5 times left leg steps first), and throwing and catching plastic ball (using a soft volleyball) or small bean bag	1 h/d, 3 d/w, 7 w	BBS, TUG, FMA, ABC	Improvement in BBS, TUG, FMA
Jarbandhan et al. (50)	30 (20/10)	Home-based, semi-supervised physiotherapy program, the home-based physiotherapy program included stair climbing, sit-to-stand exercise and walking. The physiotherapist provide weekly telephone encouragements and instructions	CT,none is given if no physiotherapy is requested by the patient	70 min/d, 3 d/w, 8 w	BBS	No significant differences were found
Wu et al. (31)	61 (30/31)	TCMeeting V6.0 plus CT. The system consists of a computer, a projector, a camera, and a data storage system. The patient installs the system on a computer at home, and the rehabilitation engineer and rehabilitation nurse perform a personalized remote rehabilitation instruction twice a week. The rehabilitation process is divided into two phases, including health education, physical strength training, balance training, breathing training, walking training and other training for patients	CT plus telephone follow-up.During the hospitalization, the patients in the control group received routine early rehabilitation guidance and routine nursing measures. The main contents were the normal limb position, bed position transfer, and joint activity maintenance training. After discharge, Patients in the control group received only routine rehabilitation and nursing measures, including dietary guidance, medication guidance, and rehabilitation guidance, which were conducted by telephone follow-up once a week. Patients can go to the rehabilitation clinic to get rehabilitation instructions as needed	0.5 h/d, 2 d/w, 12 w	BBS, TUG, FMA	Improvement in BBS,TUG,FMA
Marques-Sule et al. (32)	29 (15/14)	Wii Fit VR plus CT. Participants of VRWiiG received, in addition to conventional PT, a virtual rehabilitation program using Nintendo Wii with the Wii Remote and Wii Balance Board. The Wii Balance Board is a lightweight board that calculates the weight and pressure that is exerted on it, detecting the displacements of the pressure center, thus allowing to train balance. Each session was divided into (1) lower limb balance training (15 minutes) and (2) upper limb training (15 minutes). Each game was performed as 2 sets with a 1-minute rest interval between each game, although time for the games and the intervals were adapted to the patient's capacity	CT, including 7 different techniques based on stroke guidelines: (1) warm-up (stationary bicycle, 15 minutes); (2) mobility and strengthening lower limb exercises in supine position (3 series,15 repetitions); (3) active-assisted/passive lower and upper limb kinesiotherapy (3 series, 15 repetitions); (4) upper limb strengthening exercises using weights and elastic bands (3 series, 15 repetitions); (5) balance, stability, and coordination exercises (3 series, 15 repetitions); (6) walking reeducation exercises with emphasis inweight transfer, swing phase, step and stride length, and training with obstacles (10 minutes); (7) cool-down stretching and mobilizations of lower and upper limbs adapted to characteristics of each participant (10 min), in the university rehabilitation clinic	0.5 h/d, 2 d/w, 4 w	BBS, TUG, POMA	Improvement in BBS,TUG and POMA
Chen et al. (49)	52 (26/26)	Home-based telerehabilitation. Patients assigned to the TR group participated in rehabilitation training at home with the Telemedicine Rehabilitation System (TRS) under the therapists' guidance. The TRS consists of a therapist end, a network data system and a patient end. Therapists supervise the patients to conduct occupational therapy (OT)/physical therapy (PT) and electromyography-triggered neuromuscular stimulation (ETNS) by live video conferencing via TRS	CT, including occupational therapy (OT) and physical therapy (PT) and electromyography-triggered neuromuscular stimulation (ETNS), training in the outpatient rehabilitation department, and the training was conducted face-to-face with the rehabilitation therapists	160 min/d, 5 d/w, 2 w	FMA	Improvement in FMA

BBS, Berg Balance Scale; TUG, Timed Up and Go Test; FMA, Fugl-Meyer Assessment; TIS, Trunk Impairment Scale; S-TIS2.0, The Spanish-version of the Trunk Impairment Scale 2.0; S-PASS, Spanish-version of Postural Assessment for Stroke Patients; S-FIST, Spanish-version of Function in Sitting Test; ABC, Activities specific Balance Confidence Scale; POMA, Tinetti performance-oriented mobility assessment.

**Table 2:** Outcomes.

**Assessment scale**	**Reference**
(1) Berg balance scale (BBS)	Downs (36)
(2) The timed up and go (TUG)	Browne and Nair (37)
(3) The Spanish version of the trunk impairment scale 2.0 (S-TIS 2.0)	Cabanas-Valdés et al. (38)
(4) The Spanish version of the function in sitting test (S-FIST)	Cabanas-Valdés et al. (39)
(5) The Spanish version of the postural assessment scale for stroke patients(S-PASS)	Benaim et al. (40)
(6) Fugl-Meyer assessment (FMA)	Gladstone et al. (41)
(7) Activities specific balance confidence scale (ABC)	Powell and Myers (42)
(8) Tinetti performance-oriented mobility assessment (POMA)	Tinetti (43)
(9) Trunk impairment scale (TIS)	Verheyden et al. (44)

### Methodology quality

**Figure 2:**
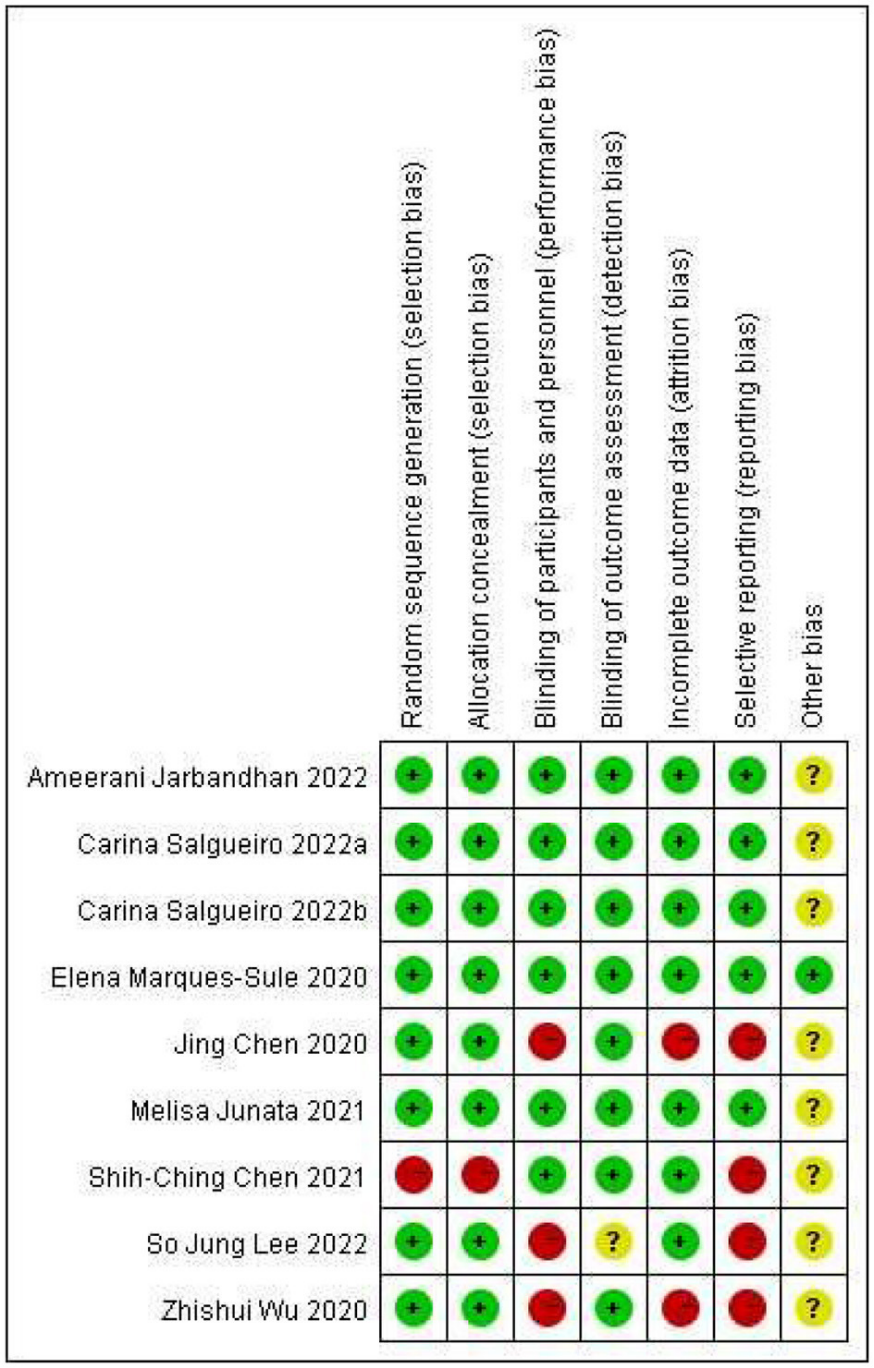
Risk-of-bias summary of all items for each included study.

**Figure 3:**
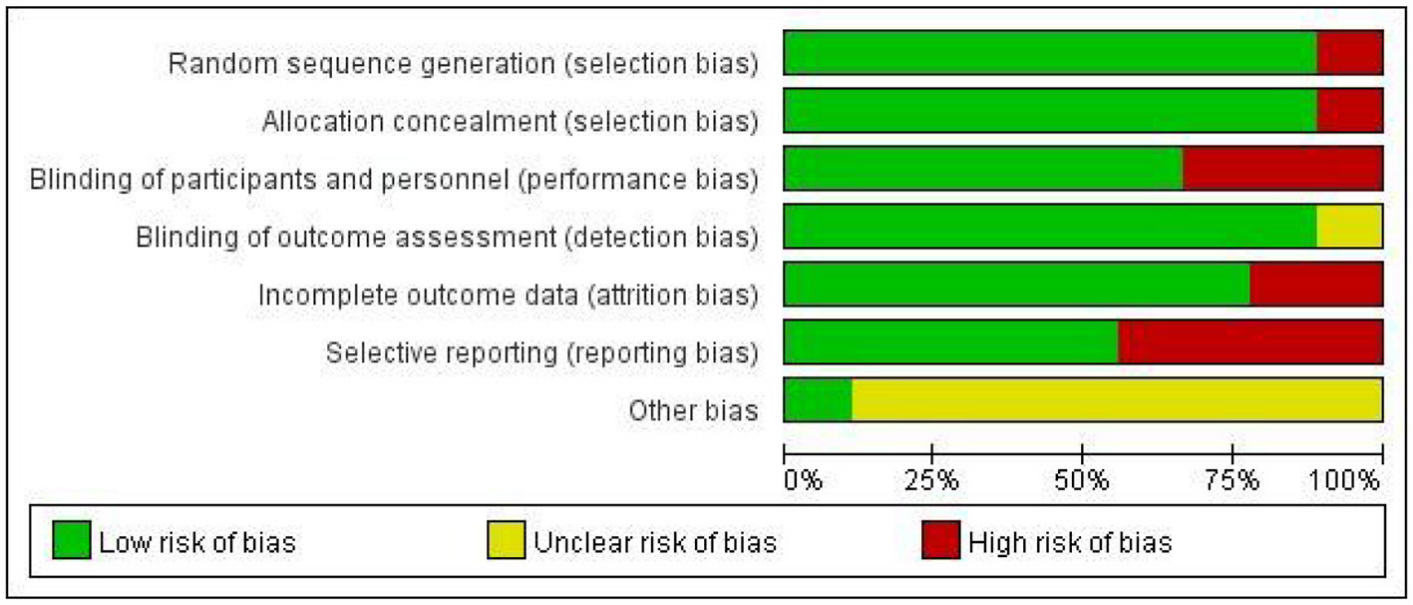
Risk-of-bias graph of all items shown as a percentage across all included studies.

The methodological quality of each study is shown in Figure 2, and all studies assessed using the Cochrane Collaboration risk-of-bias tool are shown in Figure 3. All of the studies were RCTs with clear random sequence generation and allocation except for one of the trials (30), which only reported that the patients were randomly assigned but did not specify the method of randomization or how the groups were assigned to avoid selection bias. One study (49) indicated that it was difficult to blind caregivers, therapists, and patients due to the nature of the intervention. Two studies (9, 31) did not report blinding, while the remaining studies reported well-developed blinding. Most studies had fewer data on incomplete outcomes, except for two studies (31, 49). Four studies (9, 30, 31, 49) had possible reporting bias since the study protocols were not available. In addition, they reported more outcomes, which may be associated with overreporting and a higher risk of bias. In terms of other biases, only one study (32) provided a detailed description of the aspects, including financial support, while all other studies were unclear.

### Intervention effect

#### Comparison 1: Berg balance scale. Telerehabilitation vs. conventional rehabilitation

**Figure 4:**
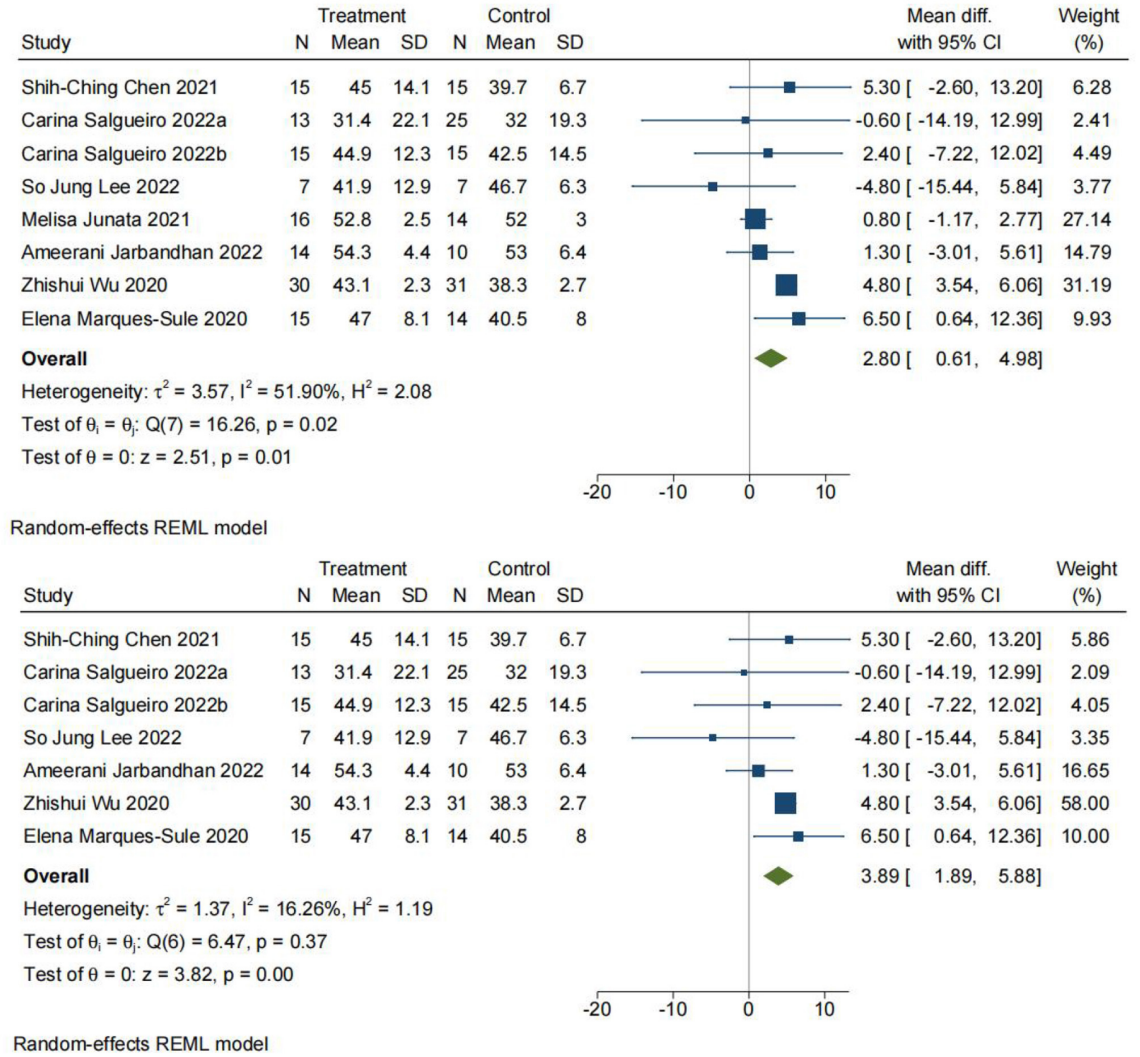
Comparison 1. Berg Balance Scale (BBS). Telerehabilitation vs. conventional rehabilitation. SD: standard deviation; 95% CI: 95% confidence interval.

**Figure 5:**
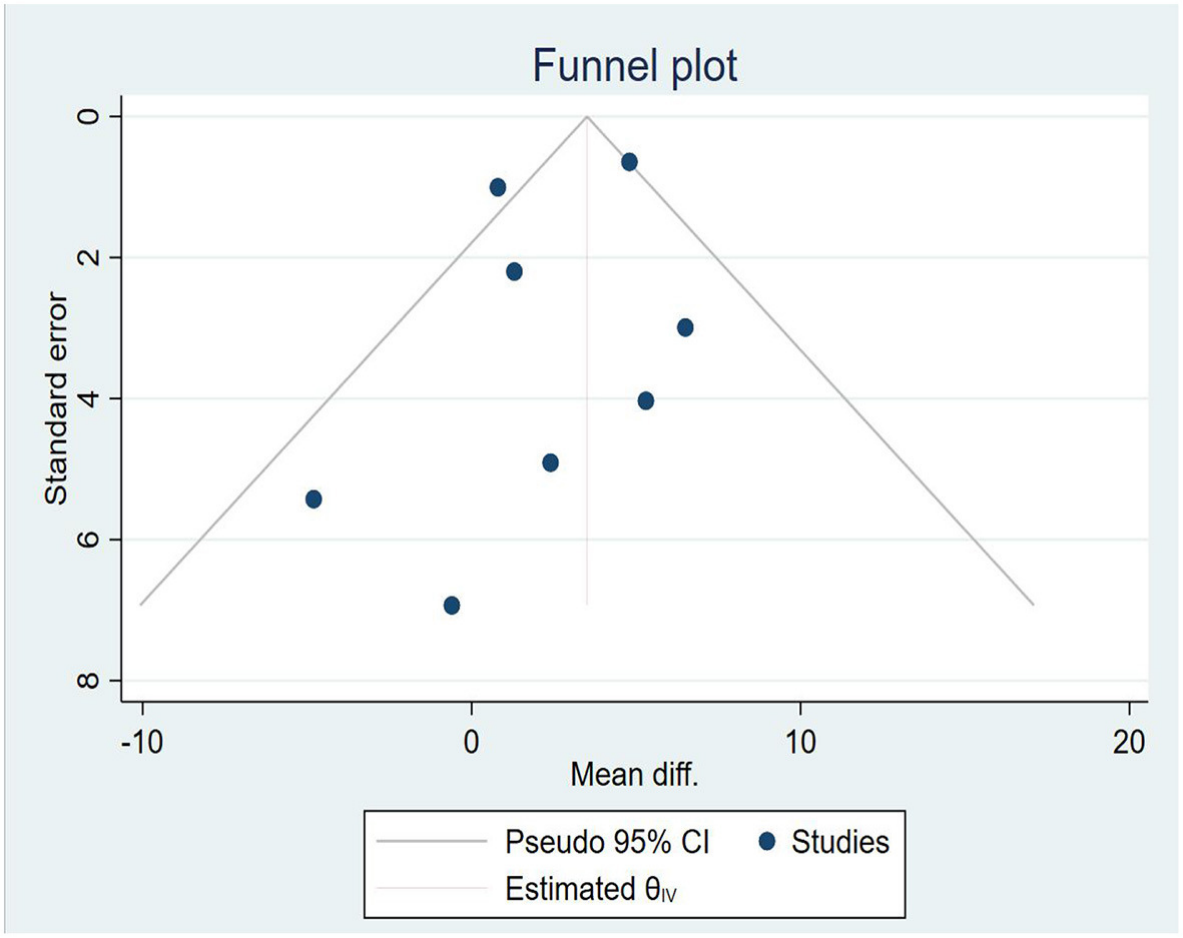
Funnel plot.

The Berg Balance Scale (BBS) is a tool globally known to evaluate balance and has been reported to be reliable and valid in cases of stroke (58, 59). The internal reliability of BBS in the elderly and stroke patients was 0.98 and 0.97, respectively (60). It consists of a series of 14 functional balance tasks to evaluate balance in different postures, including maintaining a quiet posture, sitting, transferring weight and stretching, turning in place, standing on one leg, and maintaining a tandem posture (30, 53). Each task is scored on a 5-point scale (from 0 to 4). The value 0 indicates an inability to perform the task, and the value 4 indicates the ability to complete the task according to a predetermined standard. The maximum score is 56 points. As provided in Figure 4, eight studies containing 275 patients reporting BBS were analyzed. The analysis was performed using MD with a random effects model and a confidence interval (CI) of 95%. The analysis showed a significant difference between TR and CR (MD = 2.80; 95% CI 0.61, 4.98, I2 = 51.90%). The heterogeneity of the meta-analysis was significantly reduced by sensitivity analysis after excluding the study by Melisa Junata et al. (33) from the meta-analysis. There was still a significant difference between TR and CR (MD = 3.89; 95% CI 1.89, 5.88, I2 = 16.26%), and the funnel plot is provided in Figure 5.

#### Comparison 2: timed up and go test. Telerehabilitation vs. conventional treatment

**Figure 6:**
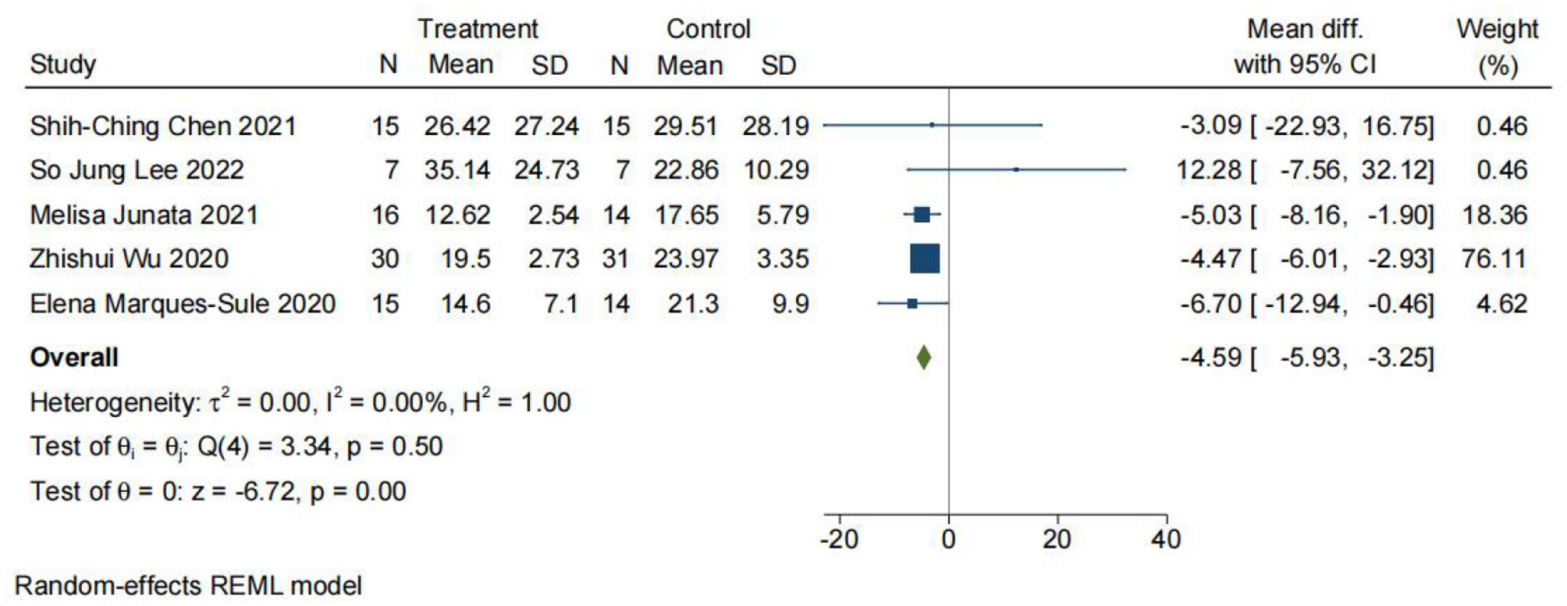
Comparison 2. Timed Up and Go (TUG) test. Telerehabilitation vs. conventional rehabilitation. SD, standard deviation; 95% CI, 95% confidence interval.

The Timed Up and Go (TUG) test is commonly used to examine functional mobility, balance, and fall risk (33, 44, 53, 61). A cone is placed 3 m from the front of the chair, and participants are asked to stand up, walk 3 m to the cone, walk around the cone, walk back, and sit down (30). The score showed excellent intra-rater, inter-rater, and retest reliability in patients with chronic stroke (62). As shown in Figure 6, a total of five studies with 167 participants were included in the meta-analysis. The analysis was performed using mean difference (MD). There was a significant difference between TR and CR for the Timed Up and Go test (MD = −4.59; 95% CI −5.93, −3.25, *I*^2^ = 0).

#### Comparison 3: the spanish version of the trunk impairment scale 2.0. Telerehabilitation vs. conventional rehabilitation

**Figure 7:**
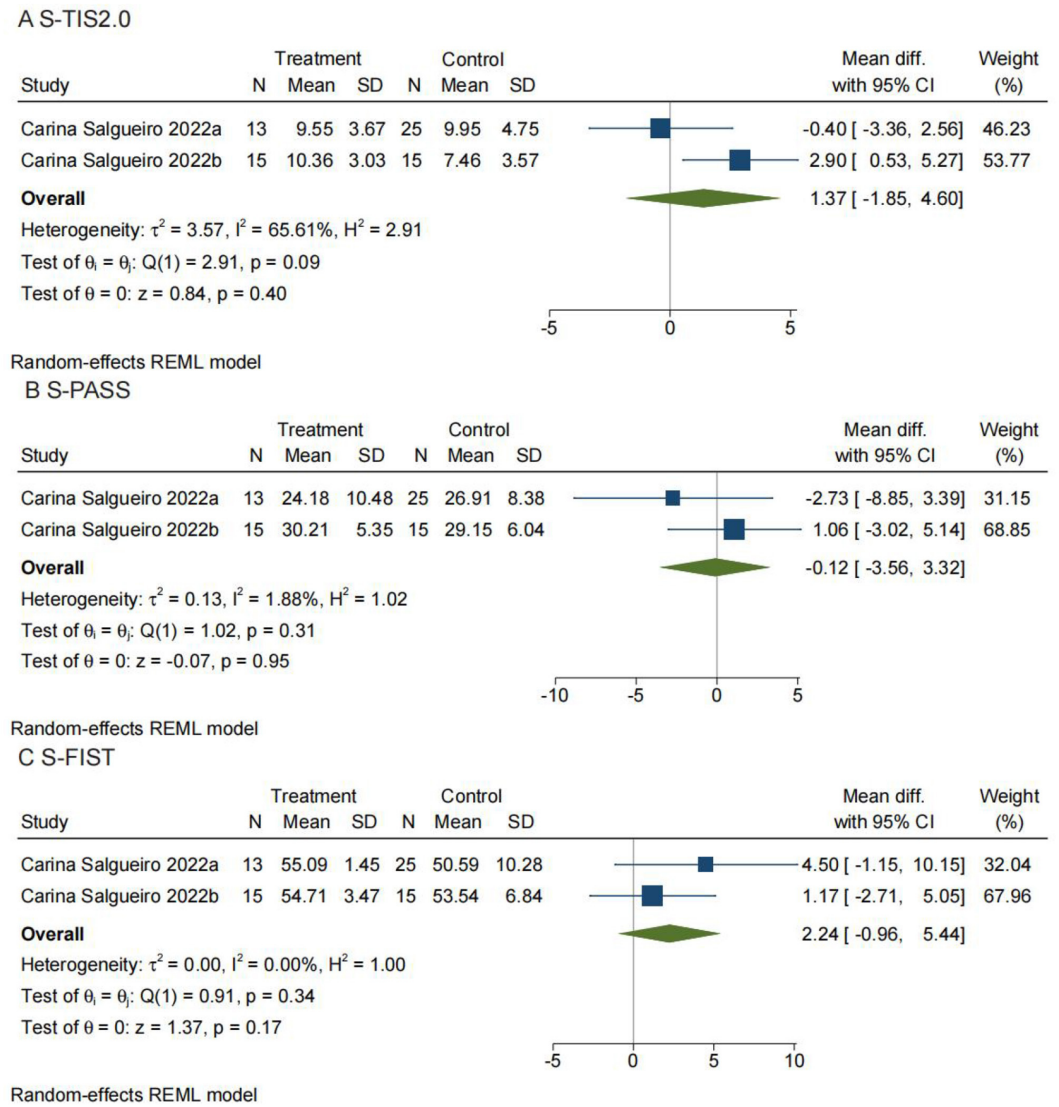
Comparisons 3, 4, and 5: **(A)** The Spanish version of the Trunk Impairment Scale 2.0 (S-TIS 2.0). Telerehabilitation vs. conventional rehabilitation. **(B)** The Spanish version of the Postural Assessment for Stroke Patients (S-PASS). Telerehabilitation vs. conventional rehabilitation. **(C)** The Spanish version of the Function in Sitting Test (S-FIST). Telerehabilitation vs. conventional rehabilitation. SD, standard deviation; 95% CI,95% confidence interval.

The S-TIS 2.0 is a clinical test that assesses movement disorders and is a reliable scale for evaluating dynamic sitting balance and trunk coordination in stroke survivors (38). As shown in Figure 7A, two studies containing 79 participants were included in the meta-analysis. Here, the analysis was also performed using MD with a random effects model, and the analysis showed no significant difference between TR and CR (MD = 1.37; 95% CI −1.85, 4.60, *I*^2^ = 65.61%).

#### Comparison 4: Spanish version of postural assessment for stroke patients. Telerehabilitation vs. conventional rehabilitation

The PASS is suitable for evaluating the postural abilities of stroke patients in the 1st months after stroke in a neurological and rehabilitation context. Among the different postural scales dedicated to stroke patients, the PASS has undergone one of the most complete validation phases (40). As shown in Figure 7B, two studies with a total of 79 participants were included in the meta-analysis. The analysis was also performed using MD with a random effects model, and the results showed no significant difference between TR and CR (MD = −0.12; 95% CI −3.56, 3.32, *I*^2^ = 1.88%).

#### Comparison 5: Spanish version of function in sitting test. Telerehabilitation vs. conventional rehabilitation

This scale consists of 14 test items corresponding to daily functional activities. Performance is scored by the therapist using a set of scoring criteria for all items. It can be used for a variety of purposes, such as evaluating functional sitting ability, describing sitting balance dysfunction, selecting the most appropriate treatment, and tracking changes in sitting balance over time (39). As shown in Figure 7C, there were also two studies with 79 participants that were included in the meta-analysis. The analysis was also performed using MD with a random effects model. No significant difference was found between TR and CR (MD = 2.24; 95% CI −0.96, 5.44, *I*^2^ = 0).

#### Comparison 6: trunk impairment scale. Telerehabilitation vs. conventional rehabilitation

**Figure 8:**
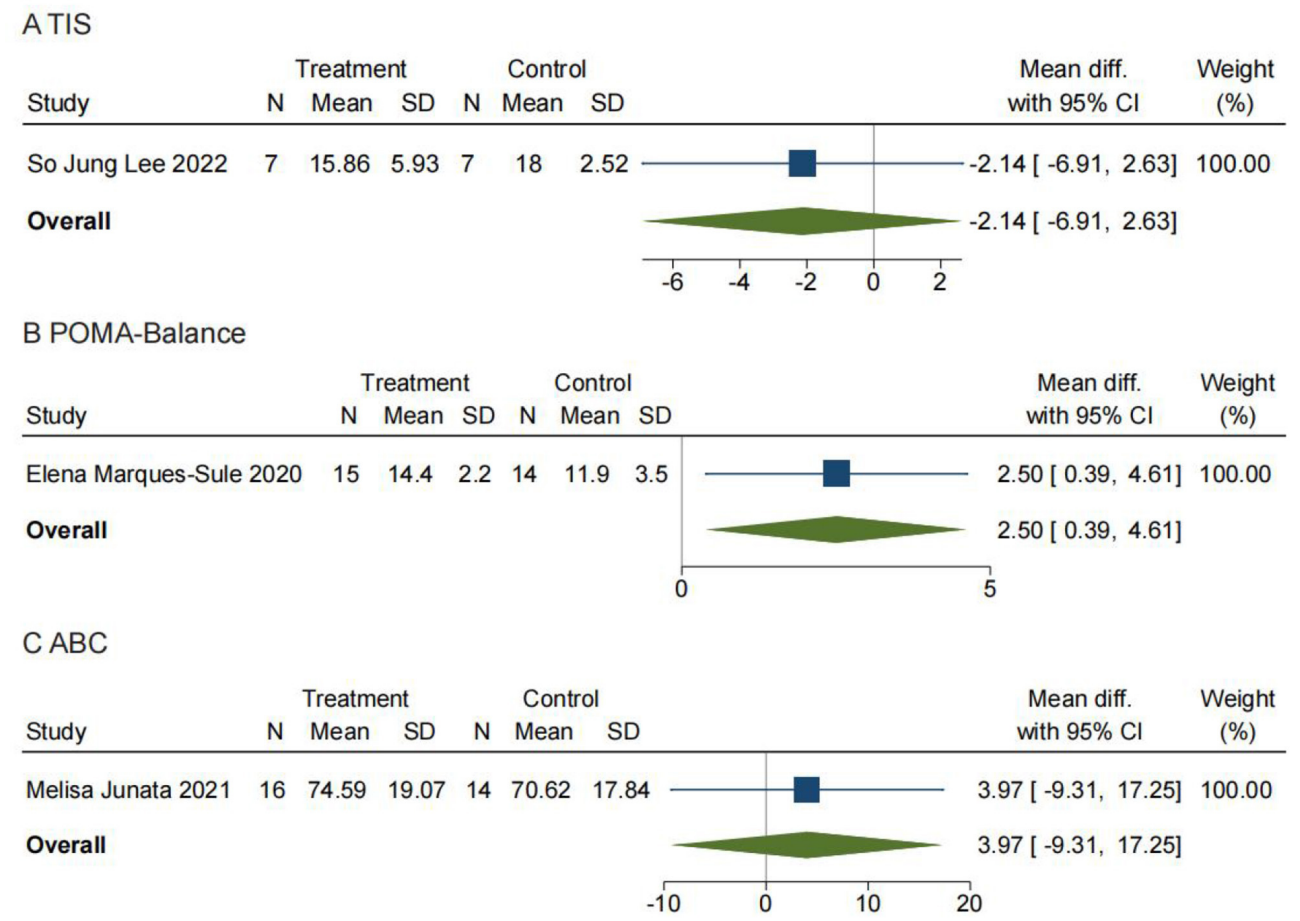
Comparisons 6, 7, and 8: **(A)** Trunk Impairment Scale (TIS). Telerehabilitation vs. conventional rehabilitation. **(B)** Tinetti performance-oriented mobility assessment—balance (POMA—Balance). Telerehabilitation vs. conventional rehabilitation. **(C)** Activities-specific balance confidence scale (ABC). Telerehabilitation vs. conventional rehabilitation. SD, standard deviation; 95% CI: 95% confidence interval.

The Trunk Impairment Scale (TIS) for patients after stroke was designed to measure ADL (activities of daily living)-related selective trunk movements rather than the participation of the trunk in gross transfer movements (44). The TIS has no ceiling effect in subacute and chronic stroke patients and already appeared to be strongly related to measures of gait, balance, and functional ability in a cross-sectional study (55, 63). As shown in Figure 8A, a total of 17 patients in one study reported TIS, with no significant difference between TR and CR (MD = −2.14; 95% CI −6.91, 2.63).

#### Comparison 7: Tinetti performance-oriented mobility assessment—Balance (POMA-Balance). Telerehabilitation vs. conventional rehabilitation

The Tinetti Performance-Oriented Mobility Assessment (POMA) is a balance tool that was originally developed for use in the institutionalized, older adult population and contains both a balance and a gait component (43). The balance component of the test assesses the patient's ability to maintain postural control while sitting statically, while rising from a chair, during the period immediately after standing, while standing with eyes open and eyes closed, while turning 360°, and during perturbation. The gait component assesses symmetry, initiation, continuity, path, the base of support, and postural sway during gait (64). The POMA measures reactive balance by asking the patient to react to a perturbation and has items such as base of support and trunk sway, which are measured during gait and are aspects of balance that are not measured by the BBS. It also evaluates step length, floor clearance, the base of support, and path deviation during gait, which is not captured in the TUG. The POMA may be a more useful measure than the BBS or TUG in patients who have dynamic balance deficits during walking or have difficulty with reactive balance (65). As shown in Figure 8B, a total of 29 patients in one study reported POMA and the balance section in it. We found a significant difference in the balance section of POMA between TR and CR (MD = 2.50; 95% CI 0.39, 4.61).

#### Comparison 8: activities-specific balance confidence scale. Telerehabilitation vs. conventional rehabilitation

The 16-item ABC scale was used to evaluate older adults' fear of falling as a response to their confidence in their balance. As shown in Figure 8C, 30 patients in one study reported ABC with no significant difference found between TR and CR (MD = 3.97; 95% CI −9.31, 17.25).

#### Comparison 9: Fugl-Meyer assessment of motor recovery after stroke. Telerehabilitation vs. conventional rehabilitation

**Figure 9:**
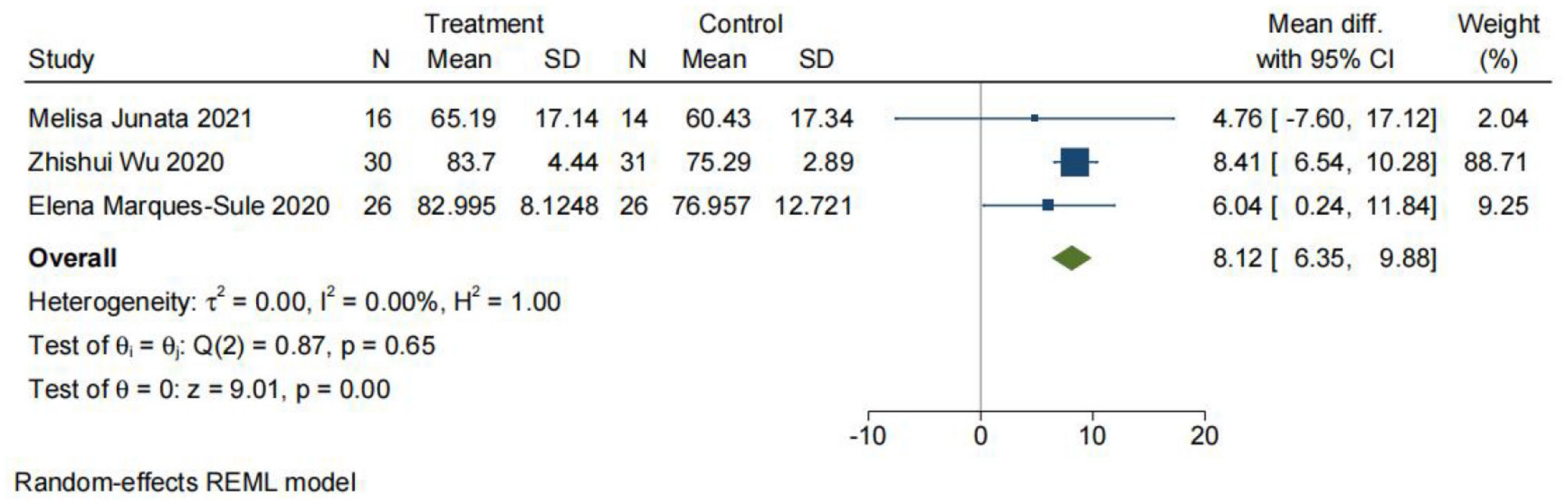
Comparison 9: Fugl-Meyer Assessment of Motor Recovery After Stroke (FMA). Telerehabilitation vs. conventional rehabilitation. SD: standard deviation; 95% CI: 95% confidence interval.

The FMA evaluates limb movement, balance, sensation, joint mobility, and pain (31) and is evaluated sequentially from the flaccid phase, the stagnation period, the combined movement period, the partial separation period, and the separation movement period based on the theory of Brunnstrom limb function recovery. Each project is divided into three levels, with 0 points (not completed), 1 point (partially completed), and 2 points (fully completed). The total score for the upper extremity is 66 points, and the total score for the lower extremity is 34 points out of 100 points (31). As shown in Figure 9, 143 patients from three studies were included in the meta-analysis, which showed a significant difference between TR and CR (MD = 8.12; 95% CI 6.35, 9.88, *I*^2^ = 0).

## Discussion

The purpose of this systematic review was to analyze and synthesize the evidence on the effectiveness of telerehabilitation interventions for patients after stroke compared to conventional face-to-face rehabilitation modalities against the background of the COVID-19 pandemic, as well as to assess which modality is more effective, telerehabilitation vs. conventional rehabilitation, to provide recommendations on the choice of rehabilitation modalities for patients after stroke during the pandemic. SARS-CoV-2 is a global pandemic that began in 2019, and the pandemic of SARS-CoV-2 was continuing until the writing of this manuscript, with mutations of the virus continuing and multiple new subtypes of the virus emerging. Each infection with a new coronavirus can be extremely damaging to the body. During the COVID-19 pandemic, patients, especially stroke patients, had limited access to outpatient rehabilitation. Telerehabilitation or the use of computer-assisted training systems can be used for stroke rehabilitation during the COVID-19 pandemic by minimizing face-to-face interactions and the risk of infection (33). We evaluated BBS, TUG, S-TIS 2.0, S-PASS, S-FIST, ABC, POMA—Balance, TIS, and FMA as outcome indicators for stroke patients, each partially reflecting the ability of patients after stroke to balance and the corresponding improvement after they have been rehabilitated. Generally, the intervention effects of the TR and CR groups were equivalent in all studies, among which BBS and FMA supported better efficacy of telerehabilitation; however, TUG and POMA—Balance supported better efficacy of the conventional rehabilitation model, with no statistical difference between the two groups in other outcomes. To the best of our knowledge, our study is the first to investigate the efficacy of telerehabilitation vs. conventional rehabilitation using data from studies conducted during the COVID-19 pandemic, and the results have been able to demonstrate that TR interventions possess the same efficacy as CR, in line with the results of previous studies (66). The coronavirus has already spread worldwide. Globally, nearly 2.8 million new cases and over 13,000 deaths were reported in the week of 9–15 January 2023. From 19 December 2022 to 15 January 2023, nearly 13 million cases and almost 53,000 new deaths were reported globally (67). The severity of the virus rampage may even be more serious since these are only the reported figures. To date, SARS-CoV-2 has a high mutation rate and continues to mutate at a rapid rate (67). In the long term, the COVID-19 pandemic will continue for a long time. Furthermore, the coronavirus may remain with humans like the influenza virus. Since stroke survivors are vulnerable, each infection may cause great harm before they fully recover. Therefore, stroke patients need to reduce any possible exposure to the virus. Telerehabilitation also plays an increasingly important role. In addition, it is important to conduct a meta-analysis utilizing data from studies published during the pandemic since patient care and access to health care have changed significantly. Additionally, it has been demonstrated that TR applies to all neurological disorders (66), and recent studies on neurological diseases also support this notion (68, 69).

With low heterogeneity, the greater improvement of BBS and FMA in the TR group compared to the CR group suggested that TR is more effective in promoting poststroke rehabilitation to some extent. The BBS and FMA scales have more content than other scales for assessing balance in sitting and standing positions and in some specific positions. Based on the content and characteristics of the BBS and FMA scales (24, 25), we suggest that telerehabilitation may be more useful for the rehabilitation of balance in sitting, standing, and specific static postures after stroke, while the rehabilitation of balance in locomotion and reactive balance is similar or more effective in the conventional modality as the TUG and POMA—Balance results from our meta-analysis support this view.

Moreover, we found that different intervention devices and their corresponding intervention modalities may have a significant impact on the results of TR intervention in patients after stroke. In two papers with the highest heterogeneity in this study (32, 33), one demonstrated an intervention based on Kinect with accompanying telerehabilitation and interactive body motion detection technology, while the other was based on Wii Fit with telerehabilitation and interactive body movement detection technology. The rest of the intervention devices and rehabilitation modalities in the TR group were customized on their own. We did not find any literature on different intervention devices and their intervention modalities during the meta-analysis. However, we may conduct corresponding research in the near future.

Finally, the question of how the effectiveness of telerehabilitation compares to that of conventional rehabilitation, which is more effective and which should be used, is no longer a question of which treatment is more appropriate for a particular disease group but rather what treatment is available to that group in the pandemic context. Since patients may not receive rehabilitation in the conventional model at all under the risk of pandemic infection and in the context of a lockdown brought about by the possible emergence of new variants of the virus (8), TR may become the only way for some patients to receive treatment and maintain their connection to society in a pandemic situation. With the further development of Internet technology and 5G, the advantages and adaptability of telerehabilitation have been increasingly reflected. In the meantime, telerehabilitation spares more healthcare expenses and allows more people to be treated while opening up a wider medical market, which is the call of the entire medical community as well as society as a whole. We have been able to demonstrate that TR can achieve similar rehabilitation results as CR. In the future, we will attempt to explore ways to achieve a better level of rehabilitation for patients through TR, which will be a focus of our study and will be beneficial to the majority of patients.

### Strengths and limitations

The strength of this study is that we carefully screened the databases to include the most relevant randomized controlled trials in an attempt to provide a strong basis for decision-making and program planning for telerehabilitation. Additionally, during our investigation, we searched for studies that are currently lacking, such as different modalities of telerehabilitation interventions that may lead to different intervention effectiveness. Besides, our study incorporated the context of the COVID-19 pandemic as people's lives after the emergence of COVID-19 have been significantly different from those before the pandemic. We also propose that the efficacy of telerehabilitation vs. conventional modalities of rehabilitation may differ across patients in terms of physical function, which is worthwhile to conduct a correspondingly detailed study. This study also has inevitable drawbacks that need to be pointed out. First, the number of included studies was small, and the lack of sample size limited further findings. In addition, only one set of data was available for some outcome measures, which may have affected our statistical analysis. Additionally, our outcome indicators are all scales. Although the scales we analyzed are all the most used scales in clinical and research practice, it is undeniable that recall bias will inevitably occur in the assessment process of scales. Avoiding such deviations completely is very difficult (7). In terms of source, five of the nine studies were conducted in China and three in Spain, both of which had serious infections during the initial outbreak of COVID-19. However, the pandemic has become a global problem, and shortly, there will be no great differences in the distribution of the pandemic worldwide. During the literature search, we also identified from the literature sources that there may be significant regional differences in the application of telerehabilitation, and therefore, there is a need to promote telerehabilitation globally, especially outside of East Asia and Europe.

## Conclusion

Telemedicine and telerehabilitation are currently gradually becoming hot topics, especially in the current COVID-19 pandemic. Admittedly, the public does not yet consider that telerehabilitation can replace the conventional modality of rehabilitation, but telerehabilitation has the potential to serve as a complement to the conventional rehabilitation modality. It can contribute to reducing the cost of rehabilitation and saving medical resources while minimizing social pressures on health care as well as extending the duration of patients' rehabilitation. Studies during the COVID-19 pandemic have demonstrated that, for stroke patients, telerehabilitation has similar efficacy to the conventional modality of rehabilitation while possibly having better efficacy in terms of static balance. However, the conventional rehabilitation modality is superior in terms of reactive balance. It is probably associated with the equipment that is used for the different interventions as well as the rehabilitation program. Meanwhile, this study revealed that more research on telerehabilitation for stroke patients and patients with other diseases is still needed and that different intervention devices and rehabilitation protocols will have to be investigated and compared. The overall quantity and quality of research still need to be improved sustainedly. Finally, it is particularly important to promote telerehabilitation worldwide, which could also contribute to the development of telerehabilitation and lead to a better prognosis for patients while providing more comprehensive and credible evidence for the study of the field.

## Data availability statement

The original contributions presented in the study are included in the article/Supplementary material, further inquiries can be directed to the corresponding author.

## Author contributions

Execution: ZS, ZG, WW, and YaoL. Aanalysis: WW, MZ, WC, HX, and NM. Design, writing and editing of the final version of the manuscript, and contributed to the article and approved the submitted version: All authors.
