# Supplementary material for: The effect of telerehabilitation on balance in stroke patients: is it more effective than the traditional rehabilitation model? A meta-analysis of randomized controlled trials published during the COVID-19 pandemic
Source: Front Neurol. 2023 May 17;14:1156473. doi: 10.3389/fneur.2023.1156473 (PMC10229885; doi:10.3389/fneur.2023.1156473)
Supplement: Supplementary file 1 [file Table_1.DOCX]

**Stroke**

Cerebrovascular Accident

Cerebrovascular Apoplexy

Brain Vascular Accident

Apoplexy

acute focal cerebral vasculopathy

brain blood flow disturbance

brain vascular accident

cerebral vascular accident

cerebrovascular arrest

cerebrovascular failure

cerebrovascular injury

cerebrovascular insult

**Tele-rehabilitation**

Tele rehabilitation

Remote Rehabilitation

Virtual Rehabilitation

Telerehabilitation

e-rehabilitation

**Pubmed**

("Cerebrovascular Accident"[Title/Abstract] OR "Cerebrovascular Apoplexy"[Title/Abstract] OR "Brain Vascular Accident"[Title/Abstract] OR "Apoplexy"[Title/Abstract] OR "acute focal cerebral vasculopathy"[Title/Abstract] OR "Brain Vascular Accident"[Title/Abstract] OR "cerebral vascular accident"[Title/Abstract] OR "cerebrovascular failure"[Title/Abstract] OR "cerebrovascular injury"[Title/Abstract] OR "cerebrovascular insult"[Title/Abstract] OR "stroke"[MeSH Terms]) AND ("Telerehabilitation"[MeSH Terms] OR ("Tele rehabilitation"[Title/Abstract] OR "Remote Rehabilitation"[Title/Abstract] OR "Virtual Rehabilitation"[Title/Abstract] OR "Telerehabilitation"[Title/Abstract] OR "e-rehabilitation"[Title/Abstract])) **52**

**Embase**

#1 'Stroke' OR 'Cerebrovascular Accident' OR 'Cerebrovascular Apoplexy' OR 'Brain Vascular Accident' OR 'Apoplexy' OR 'acute focal cerebral vasculopathy' OR 'brain blood flow disturbance' OR 'brain vascular accident' OR 'cerebral vascular accident' OR 'cerebrovascular arrest' OR 'cerebrovascular failure' OR 'cerebrovascular injury' OR 'cerebrovascular insult'

#2 'Tele-rehabilitation' OR 'Tele rehabilitation' OR 'Remote Rehabilitation' OR 'Virtual Rehabilitation' OR 'Telerehabilitation' OR 'e-rehabilitation'

#3 #1 AND #2 **106**

**Web of Science**

#1 TS=（"Stroke" OR "Cerebrovascular Accident" OR "Cerebrovascular Apoplexy" OR "Brain Vascular Accident" OR "Apoplexy" OR "acute focal cerebral vasculopathy" OR "brain blood flow disturbance" OR "brain vascular accident" OR "cerebral vascular accident" OR "cerebrovascular arrest" OR "cerebrovascular failure" OR "cerebrovascular injury" OR "cerebrovascular insult"）

#2 TS=（"Tele-rehabilitation" OR "Tele rehabilitation" OR "Remote Rehabilitation" OR "Virtual Rehabilitation" OR "Telerehabilitation" OR "e-rehabilitation"）

#3 #1 AND #2 219

**Cochrane Library**

#1 MeSH descriptor: [Stroke] explode all trees 14102

#2 (Cerebrovascular Accident):ti,ab,kw OR (Cerebrovascular Apoplexy):ti,ab,kw OR (Brain Vascular Accident):ti,ab,kw OR (Apoplexy):ti,ab,kw OR (acute focal cerebral vasculopathy):ti,ab,kw 16229

#3 #1 OR #2 27258

#4 MeSH descriptor: [Telerehabilitation] explode all trees 229

#5 (Tele rehabilitation):ti,ab,kw OR (Remote Rehabilitation):ti,ab,kw OR (Virtual Rehabilitation):ti,ab,kw OR (Tele-rehabilitation):ti,ab,kw OR (e-rehabilitation):ti,ab,kw 2382

#6 #4 OR #5 2527

#7 # AND #6 **451**
